# Activation of the PERK/eIF2α axis is a pivotal prerequisite of taxanes to cancer cell apoptosis and renders synergism to overcome paclitaxel resistance in breast cancer cells

**DOI:** 10.1186/s12935-024-03443-w

**Published:** 2024-07-17

**Authors:** Wanhua Cai, Dade Rong, Jiayu Ding, Xiaomei Zhang, Yuwei Wang, Ying Fang, Jing Xiao, Shulan Yang, Haihe Wang

**Affiliations:** 1 Center for Translational Medicine, the First Affiliated Hospital, Sun Yat-sen University, 58 Second Zhongshan Road, Guangzhou, 510080 China; 2 Department of Biochemistry, Zhongshan School of Medicine, Sun Yat-sen University, 74 Second Zhongshan Road, Guangzhou, 510080 China; 3 Faculty of Health Sciences, University of Macau, Taipa, Macau SAR China; 4 School of Medicine, Xizang Minzu University, No.6 Wenhui Donglu, Xianyang, 712082 China; 5 Department of Clinical Laboratory, Zhuhai Interventional Medical Center, Zhuhai Precision Medical Center, Zhuhai People’s Hospital (Zhuhai Hospital Affiliated with Jinan University), Zhuhai, 519000 China; 6 Clinical Medical Research Centre for Plateau Gastroenterological Disease of Xizang Autonomous Region, Xizang Minzu University, Xianyang 712082, China

**Keywords:** Taxane, PERK/eIF2α, Tumor drug resistance, Breast cancer, ER stress

## Abstract

**Background:**

Microtubule polymerization is usually considered as the upstream of apoptotic cell death induced by taxanes, but recently published studies provide more insights into the mechanisms responsible for the antineoplastic effect of taxanes. In this study, we figure out the role of the stress-related PERK/eIF2α axis in tumor cell death upon taxane treatment along with paclitaxel resistance.

**Methods:**

Utilizing immunoblot assay, the activation status of PERK-eIF2α signaling was detected in a panel of cancer cell lines after the treatment of taxanes. The causal role of PERK-eIF2α signaling in the cancer cell apoptosis induced by taxanes was examined via pharmacological and genetic inhibitions of PERK. The relationship between microtubule polymerization and PERK-eIF2α activation was explored by immunofluorescent and immunoblotting assays. Eventaually, the combined therapeutic effect of paclitaxel (PTX) and CCT020312, a PERK agonist, was investigated in PTX-resistant breast cancer cells in vitro and in vivo.

**Results:**

PERK-eIF2α axis was dramatically activated by taxanes in several cancer cell types. Pharmacological or genetic inhibition of PERK efficiently impaired taxane-induced apoptotic cell death, independent of the cellular microtubule polymerization status. Moreover, PTX was able to activate the PERK/eIF2α axis in a very low concentration without triggering microtubule polymerization. In PTX-resistant breast cancer cells, the PERK/eIF2α axis was attenuated in comparison with the PTX-sensitive counterparts. Reactivation of the PERK/eIF2α axis in the PTX-resistant breast cancer cells with PERK agonist sensitized them to PTX in vitro. Combination treatment of the xenografted PTX-resistant breast tumors with PERK agonist and PTX validated the synergic effect of PTX and PERK activation in vivo.

**Conclusion:**

Activation of the PERK/eIF2α axis is a pivotal prerequisite of taxanes to initiate cancer cell apoptosis, which is independent of the well-known microtubule polymerization-dependent manner. Simultaneous activation of PERK-eIF2α signaling would be a promising therapeutic strategy to overcome PTX resistance in breast cancer or other cancers.

**Supplementary Information:**

The online version contains supplementary material available at 10.1186/s12935-024-03443-w.

## Introduction

Taxanes, including paclitaxel (PTX), docetaxel (DOC), and albumin-bound PTX (nab-PTX), are widely used as anti-cancer drugs in the clinical treatment of various solid tumors, and the mechanisms of these reagents in eradicating cancer cells has been elucidated [1–4]. As antimitotic, it is well known that these chemicals inhibit the depolymerization of microtubules by directly binding to β-tubulin, leading to M phase arrest and ultimate cell apoptosis [5–9]. Despite the successful usage of taxanes in tumor therapy, acquired resistance still compromises their clinical application [10]. Among the known mechanisms that mediate the resistance of taxanes, the overexpression of efflux pump proteins including ABCB1 and ABCG2, the upregulation of βIII-tubulin (TUBB3) and mutations at the PTX-binding site of β-tubulin are the three most common causes for taxane resistance [11–13]. Although the combination treatment of ABCB1 inhibitors with other chemotherapeutic agents displays wishful killing efficacy to a series of drug-resistant cancer cells with overexpression of ABCB1/ABCG2 in tumor models, the clinical trials results of ABCB1 inhibitors still are discouraging due to its nonspecific cytotoxicity to normal tissues [14, 15]. Moreover, there are no applicable and effective therapeutic strategies used to treat tumors with upregulation of βIII-tubulin (TUBB3) or mutations at the PTX-binding site of β-tubulin [16, 17]. In addition, it remains ambiguous whether stabilized microtubule polymerization induced by taxanes is the key or the only cause of apoptosis [18, 19]. Thus, uncovering the detailed and novel mechanisms of taxane resistance is the prerequisite for developing promising therapeutic strategies and novel drugs in clinical practice.

PERK/eIF2α axis, one of the three important pathways of unfolded protein response (UPR), is triggered by unfolded or misfolded proteins accumulating in the endoplasmic reticulum (ER) cavity after pharmacological or physiological disruption of ER folding events [20, 21]. ER stress is hinted to be involved in PTX-induced apoptosis [22]. More recently, it has been shown that the cytotoxic effects of taxanes are in part mediated by ER stress, instead of their antimitotic function [23–25].

In our previous studies, PTX and the novel taxane Difluorovinyl-ortataxel (DFV-OTX) markedly upregulated p-eIF2α in breast cancer cells. Inhibition of eIF2α phosphorylation by PERK inhibitors blocked taxane-induced H2A.X phosphorylation, a symbol of DNA double-strand break [26], emerged an important role of PERK/eIF2α axis in taxane-induced apoptosis. In this study, we used various types of cancer cells to verify whether the activation of the PERK/eIF2α axis is a general phenomenon under taxanes treatment and further explored the role of PERK/eIF2α axis in PTX resistance by using the PTX-resistant breast cancer cells. We disclosed that activation of the PERK/eIF2α axis is a novel manner of taxane action, and its dysfunction was demonstrated to be involved in the resistance of PTX. In addition, the combination of PERK agonists with PTX provides a promising therapeutically synergic strategy to overcome PTX resistance in breast cancer cells.

## Materials and methods

### Taxanes and reagents

Paclitaxel (PTX) was purchased from Selleck (Shanghai, China). Docetaxel (a semisynthetic derivative of paclitaxel), CCT020312 (a selective PERK/ eIF2α activator), two specific PERK inhibitors GSK2606414 and GSK2656157, and Z-VAD-FMK (a well-known pan-caspase inhibitor) were purchased from MCE (Shanghai, China). The Annexin V-FITC/PI double-staining Kit was purchased from BestBio Biotechnology (Guangzhou, China). Difluorovinyl-ortataxel (DFV-OTX) was synthesized and fully characterized as previously described [26].

### Cell lines and cell culture

HCT-116, MCF-7and MDA-MB-231 cells were obtained from the American Tissue Culture Collection (USA) and cultured in RPMI 1640 (HyClone, USA) with 10% FBS (Gibco, USA) and 1% antibiotics (Penicillin/Streptomycin, HyClone, USA). A549 and 293T cells were respectively provided by Dr. Cai and Dr. Li from Sun Yat-sen University, China, and cultured in DMEM with 10% FBS and 1% antibiotics. PTX-resistant MDA-MB-231 cells (MDA-MB-231R) were provided by Dr. Yang from Jinan University, China. PTX-resistant MCF-7 cells (MCF-7R) were established as previously described [26]. MCF-7R cells and MDA-MB-231R cells were respectively grown in DMEM with 10 nM or 100 nM PTX along with 10% FBS and 1% antibiotics. All cells were cultured in 5% CO_2_ at 37 °C.

### Immunofluorescence staining

The cells were seeded into six-well plates with cover slips in each well, and then treated with PTX combined with or without PERK inhibitors for 24 h. After fixing with 4% PFA for 20 min and blotting with 5% BSA for 1 h, cells were subsequently incubated with anti-rabbit β-tubulin primary antibody (1:100) at 4 °C overnight, followed by incubation with donkey anti-rabbit IgG conjugated to Alexa Fluor 488 secondary antibody (1:300) for 1 h. Stained cells were mounted with a mounting medium containing DAPI for 5 min and visualized under confocal microscopy.

### Cell counting kit-8 assay

Assayed cells were suspended in 100 µL culture medium and inoculated in a 96-well plate (1500 cells per well). Following 24-hour (h) incubation, cells were treated with different concentrations of PTX or CCT for 120 h. Subsequently, the plates were rinsed and incubated with fresh culture medium containing 10 µL cell counting kit-8 reagent (Dojindo Laboratories, Japan) for another 1 h. The absorbance at 450 nm wavelength was measured using a microplate reader. Cell growth inhibition of 50% (IC_50_) was calculated by GraphPad Prism 7.0 software. All assays were performed in triplicates.

### Apoptosis analysis

Cells were first plated and cultured for 24 h, PTX or CCT were then added for another 24 h. These cells were harvested by digestion with EDTA-free trypsin and carefully collected together with the floating cells in the culture plates. Cell pellets were washed once with PBS and resuspended in binding solution, followed by incubation with Annexin V-FITC on ice for 15 min, and PI for another 5 min, before analysis by flow cytometry. All assays were performed in triplicates.

### Immunoblots analysis

Floating cells in the plates were collected through a centrifuge and combined with the scrapped cells later. The left adhesion cells were rinsed with cold PBS and then scrapped off in cell lysis buffer (62.5 mM Tris-HCl with pH 6.8, 20 mg/ml SDS, 20% Glycerol), with 1% PMSF and 1% phosphatase cocktail inhibitors (CWBIO, Jiangsu, China). The whole cell lysates were subjected to SDS-PAGE. The detailed immunoblotting procedures had been described previously [26]. The relative protein expression was validated by densitometry and ImageJ software, and normalized against housekeeping protein. The relative expression of phosphorylated protein is calculated according to the normalized intensity of phosphorylated protein against the correspondingly normalized total protein amounts. Antibodies for β-actin and GAPDH detection were bought from Proteintech (USA), PERK, eIF2α, p-eIF2α, PARP, cleaved-caspase7, p-H2A.X, ATF4, caspase4 antibodies were bought from Cell Signaling Technology (USA), p-PERK and CHOP were bought from Santa Cruz Biotechnology (USA).

### Viral transduction and establishment of the stable PERK-knockdown cells

HEK-293T cells were seeded on a 6 cm culture dish with 50% confluency. After culturing for 18 h, cells were co-transfected with the lentiviral expression plasmid (pLKO.1-shPERK, 3.5 µg), viral packaging plasmid (psPAX2, Addgene 12260, 2.5 µg), and envelope plasmid (VSVG, Addgene 14888, 1µg), using PEI following the manufacturer’s protocols. Viral supernatant was collected at 48 h post-transfection and passed through a 0.45 µm sterile filter. For transduction, assay cells were seeded on a 3 cm culture dish and incubated with media supplemented with viral supernatant. After 12–24 h incubation, the virus-containing medium was replaced with a fresh culture medium. Transduced cells were selected with an optimized concentration of puromycin for 3 weeks at 48 h post-transduction. Immunoblots was performed to validate the knockdown efficiency of PERK expression. Sequences of the specific PERK shRNAs are 5’-ATCATAGCAACAACGTTTATT-3’ (PERK-shRNA1) and 5’-TTTGTCCCTGGCGGGTAAATT-3’ (PERK-shRNA2).

### Xenograft tumor experiments

MCF-7R or MDA-MB-231R cells (1 × 10^7^ cells in 150 µL PBS) were subcutaneously inoculated into the flanks of the 5-week-old female BALB/c nude mice (Laboratory Animal Center, Sun Yat-sen University). Till subcutaneous tumors formed, mice were tumor size-matched and randomly assigned to different groups for experiments. Tumor sizes were measured 2–3 times per week. Tumor volume (mm^3^) = (length×width^2^)/2. At the end of the experiments, mice were sacrificed, and tumors were dissected to examine the therapy’s efficacy. All animal studies were applied and approved by the Institutional Animal Care and Use Committee (IACUC) of Sun Yat-sen University, China.

### Statistical analysis

Statistical significance was examined using unpaired, two-tailed Student’s t-tests in GraphPad Prism 7.0 software. The data are presented as mean ± SEM. Differences were considered to be statistically significant with *p* < 0.05.

## Results

### Taxanes activate PERK/eIF2α axis in various cancer cells

**Fig. 1:**
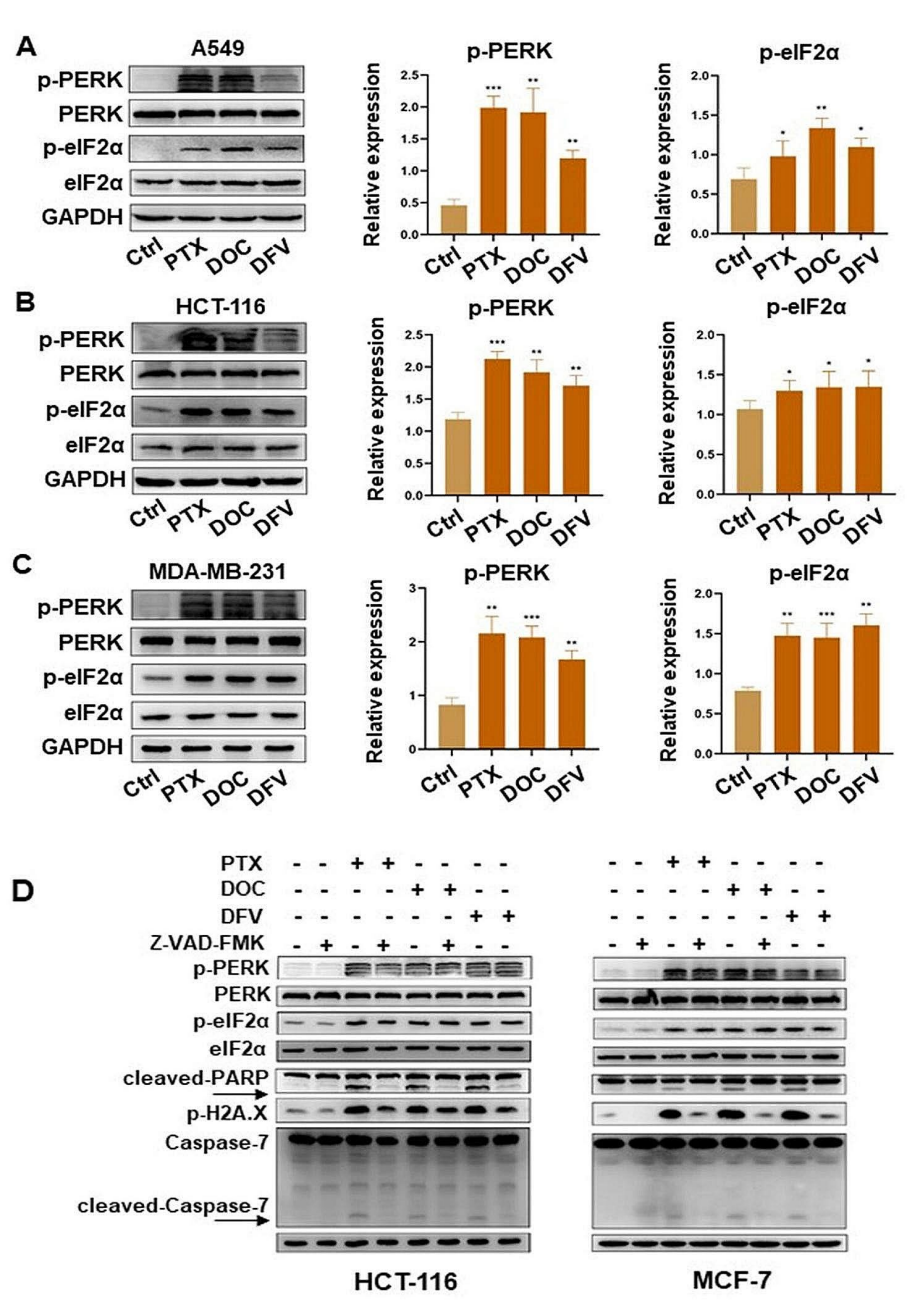
Taxanes activate PERK/eIF2α axis in various types of cancer cells

As we previously found that PTX and a novel taxane DFV-OTX both activate PERK/eIF2α to induce apoptosis in breast cancer cells [26], we sought to verify whether there is a general role of a broad spectrum of taxanes in killing cancer cells via PERK/eIF2α axis. Three taxanes, including PTX, DOC, DFV-OTX, were applied to treat different types of tumor cells. Immunoblots showed that the activation of PERK/eIF2α axis could be evidently observed in all assayed cells after the treatment of taxanes, basing on their phosphorylation levels (Fig. 1A-C). In contrast, no obvious changes were observed in the other well-known UPR-related protein in MCF-7 cells treated with PTX, including p-IRE1α and cleaved-ATF6, the activated forms of UPR sensors IRE1α and ATF6, respectively (Fig S1A). Thapsigargin (TG), a well-known ER stress inducer, was used as a positive control and it significantly increased the phosphorylation of IRE1α and slightly increased the cleavage of ATF6 (Fig S1A). Similarly, even with the high dosage of taxane (10 µM) treatment, only TG rather the three taxanes strikingly up-regulated the expression of p-IRE1α in HCT-116 cells and MDA-MB-231 cells (Fig S1B-D).

Apoptosis is the major type of cell death induced by taxanes [9, 19]. To clarify the relationship between cell apoptosis and PERK/eIF2α axis activation, HCT-116 and MCF-7 cells were treated with a panel of taxanes with or without Z-VAD-FMK, a pan-caspase inhibitor [27, 28]. Immunoblotting results showed that the addition of Z-VAD-FMK significantly inhibited the activation of Caspase-7, reduced the production of cleaved PARP and the phosphorylation of H2A.X (Fig. 1D), which indicated Z-VAD-FMK effectively abolished the apoptotic cell death induced by different taxanes. Nevertheless, striking p-PERK and p-eIF2α could still be observed when the caspase-dependent apoptosis was blocked (Fig. 1D). Together, these results suggested that taxanes particularly activate PERK/eIF2α axis and the activation of PERK/eIF2α signaling may be the upstream of apoptosis induced by taxanes.

(A-C) Immunoblots of PERK/eIF2α axis and the relative expression of p-PERK/ p-eIF2α in A549, HCT-116 and MDA-MB-231 cells treated with 100 nM taxanes (PTX, DOC, DFV-OTX) for 24 h. All assays were performed in triplicates.* *p* < 0.05; ** *p* < 0.01; *** *p* < 0.001. (D) Immunoblots of PERK/eIF2α axis and apoptosis-related proteins in HCT-116 and MCF-7 cells treated with 100 nM taxanes (PTX, DOC, DFV-OTX) combined with or without 20 µM Z-VAD-FMK for 24 h.

### Inhibition of PERK/eIF2α axis blocks taxane-induced apoptotic cell death

**Fig. 2:**
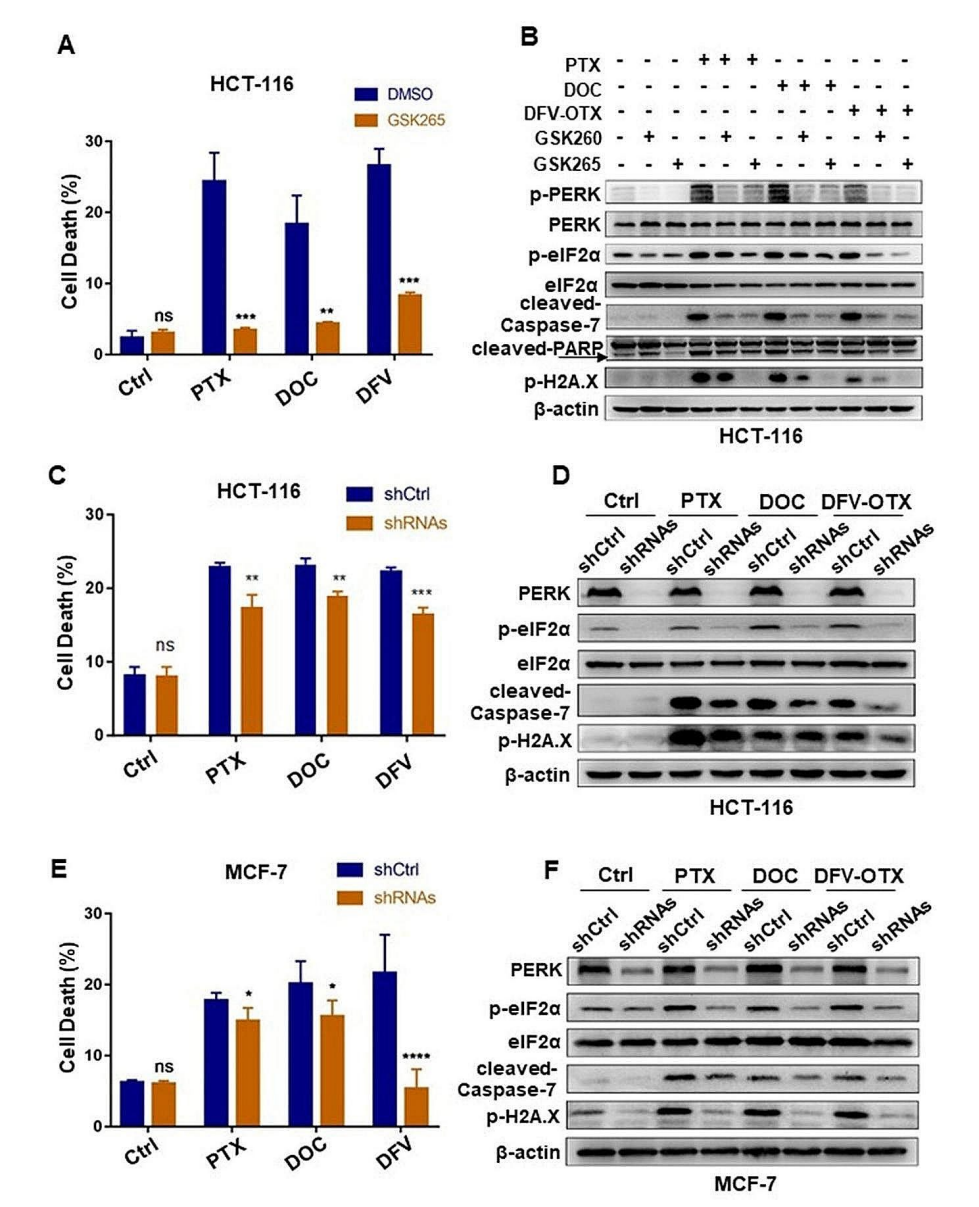
Inhibition of PERK/eIF2α axis blocks taxane-induced apoptotic cell death

To explore whether the activation of PERK/eIF2α axis is the leading cause of the apoptotic cell death induced by taxanes, we first inhibited PERK activation with two specific PERK inhibitors GSK2656157 (GSK265) and GSK2606414 (GSK260). The results of Annexin V-FITC/PI double staining assay manifested that both GSK265 and GSK260 could markedly block the taxanes-induced cell death in HCT-116 cells (Fig. 2A). Consistently, the addition of PERK inhibitors strikingly inhibited the activation of Caspase-7, reduced the cleavage of PARP and decreased the accumulation of p-H2A.X in HCT-116 cells and MCF-7 cells (Fig. 2B; Fig S2A).

To exclude the unknown non-specific effect of PERK inhibitors, we further knocked down PERK with its specific short hairpin RNAs (shRNA) to impede PERK/eIF2α axis. Annexin V-FITC/PI double staining demonstrated that knockdown of PERK similarly abrogated taxane-induced cell death in both HCT-116 and MCF-7 cells (Fig. 2C, E; Fig S2B, C). Immunoblots also confirmed the attenuated expression of the apoptosis-activating markers in PERK knockdown cells (Fig. 2D, F). Therefore, PERK/eIF2α axis activation would be an essential prerequisite for taxanes to render tumor cell apoptosis.

- (A) Percentage of cell death (Annexin V-positive) after treatment of HCT-116 cells with 100 nM taxanes (PTX, DOC, DFV-OTX) combined with 10 µM GSK265 for 24 h. All assays were performed in triplicates. ns: no statistical significance; ** *p* < 0.01; *** *p* < 0.001. (B) Immunoblots of PERK/eIF2α axis and apoptosis-related protein in HCT-116 cells treated with 100 nM taxanes combined with 10 µM GSK265 or GSK260 for 24 h. (C) Percentage of cell death (Annexin V positive) after treatment of HCT-116 cells with PERK knockdown with 100 nM taxane treatment for 24 h. All assays were performed in triplicates. ns: no statistical significance; ** *p* < 0.01, **** *p* < 0.0001.
- (D) Immunoblots of PERK/eIF2α axis and apoptosis-related protein in HCT-116 cells treated as in (C). (E) Percentage of cell death (Annexin V-positive) after treatment of MCF-7 cells with PERK knockdown with 100 nM taxane treatment for 24 h. All assays were performed in triplicates. ns: no statistical significance; * *p* < 0.05; **** *p* < 0.0001.
- (F) Immunoblots of PERK/eIF2α axis and apoptosis-related protein in MCF-7 cells treated as in (E).

### PERK/eIF2α/CHOP axis contributes to PTX-induced cell apoptosis

**Fig. 3:**
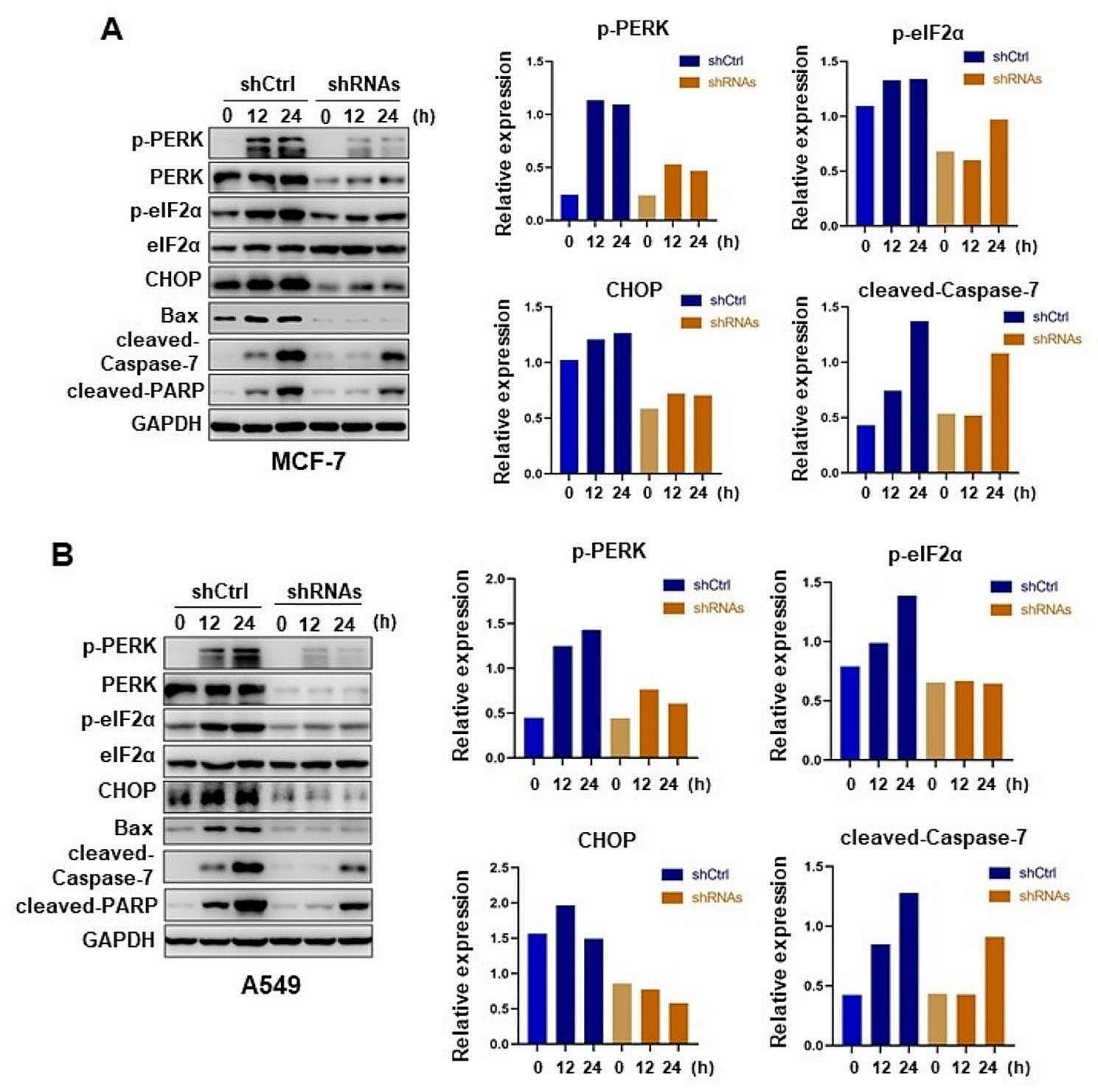
Immunoblots of the dynamic PERK/eIF2α/CHOP axis under PTX treatment

To dissect the downstream molecular events of the PERK/eIF2α axis in PTX-induced apoptosis, we performed a time course analysis of PERK/eIF2α and apoptosis pathways in MCF-7 and A549 cells. We found CHOP, a transcriptional factor that is considered a trigger of stress-induced apoptosis [29–32], is upregulated up to 24 h under 100 nM PTX treatments. In corresponding PERK-deficient cells, the induction of CHOP is nearly attenuated, followed by the lower expressions of cleaved PARP and cleaved caspase-7 (Fig. 3A, B). These results indicated that PERK/eIF2α/CHOP plays an important role in PTX-induced apoptosis.

In addition, considering caspase-4 has been implicated in ER stress-induced apoptosis [33–35], we detected its expression but found no change under up to 10 µM taxane stimulation (Fig S3), indicating caspase-4 may not involved in taxane-induced cell apoptosis.

(A-B) Immunoblots and a bar graph of the respective blots of PERK/eIF2α axis and related downstream apoptotic signal in MCF-7 cells (A) and A549 cells (B) with or without PERK-knockdown under treatment with 100 nM PTX at indicated time points. The relative expression of p-PERK, CHOP, and cleaved-Caspase-7 was normalized with the respective loading control (GPADH). The relative p-eIF2α expression is normalized against eIF2α intensities.

### Taxane-induced PERK/eIF2α axis is independent of cellular microtubule polymerization

**Fig. 4:**
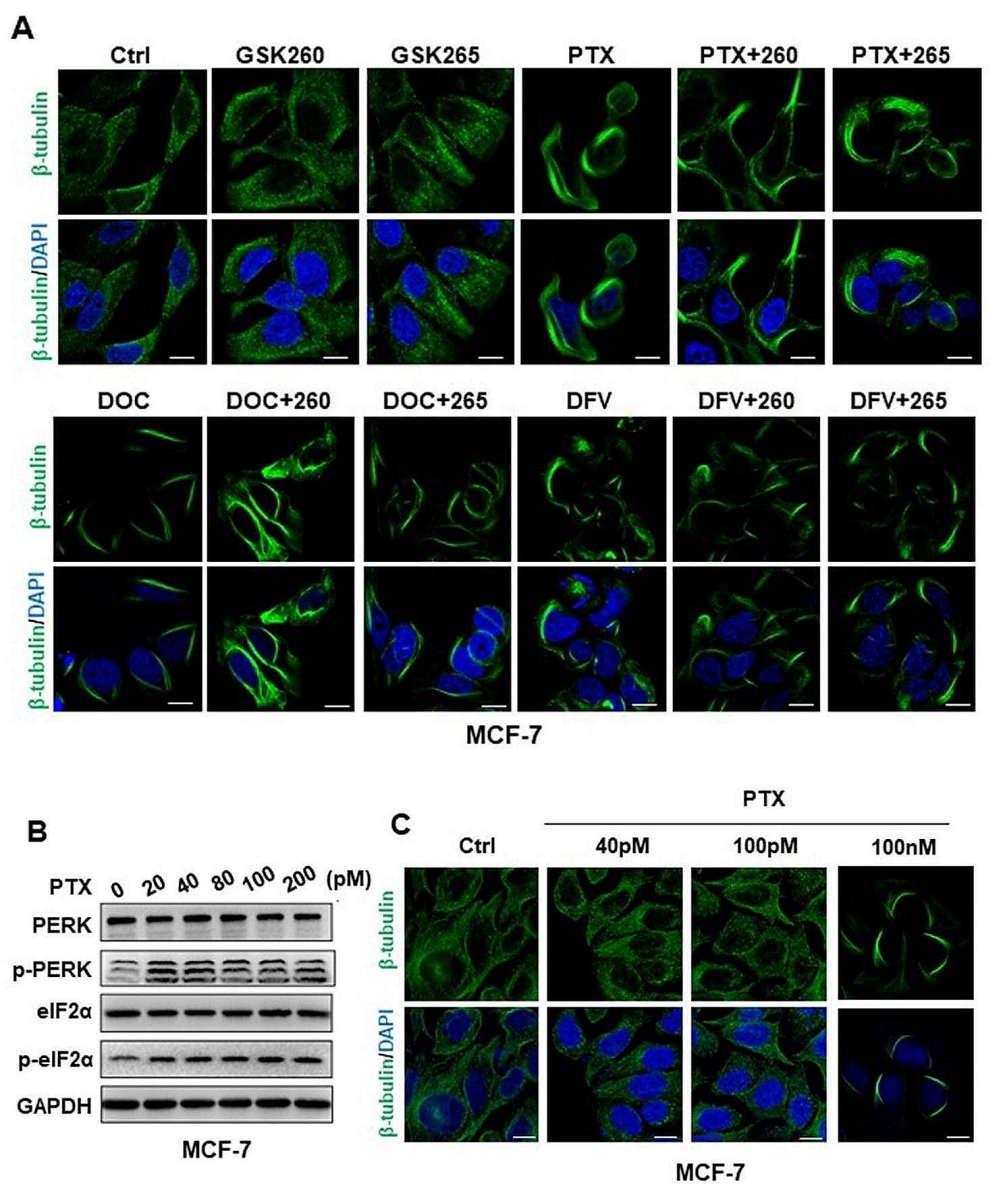
Taxanes-induced PERK/eIF2α axis is independent of cellular microtubule polymerization

Given that taxanes are anti-microtubule agents, to investigate whether the activated PERK/eIF2α axis was attributed to microtubule polymerization, we treated MCF-7 cells with taxanes combined with or without the two PERK inhibitors, GSK265 and GSK260, and performed immunofluorescence assay. Results showed that inhibition of the PERK/eIF2α axis by GSK265 and GSK260 did not weaken the microtubule polymerization induced by taxanes (Fig. 4A), suggesting PERK/eIF2α axis activation is the independent or downstream event of microtubule aggregation. Moreover, we treated cells with PTX in a very low concentration and observed evident expression of p-PERK and p-eIF2α (Fig. 4B; Fig S4A) without notable microtubule polymerization (Fig. 4C;Fig S4B), verifying the independent PERK/eIF2α axis activation of cellular microtubule polymerization upon taxane stimulation.

(A) Confocal microscopy of microtubule distribution in MCF-7 cells treated with 100 nM taxanes (PTX, DOC, DFV-OTX) combined with 10 µM GSK265 or GSK260 for 24 h. Green: β-tubulin labeled by a fluorescent antibody; blue: nuclei stained with DAPI. The indicated bars are 10 μm. (B) Immunoblots of PERK/eIF2α axis in MCF-7 cells treated with PTX at ultra-low concentration as indicated. (C) Confocal microscopy of microtubule distribution in MCF-7 cells treated with 40 pM or 100 pM PTX for 24 h. Treatment of 100 nM PTX is the positive control of microtubule polymerization.

### Reactivation of PERK effectively reverses PTX resistance in breast cancer cells

**Fig. 5:**
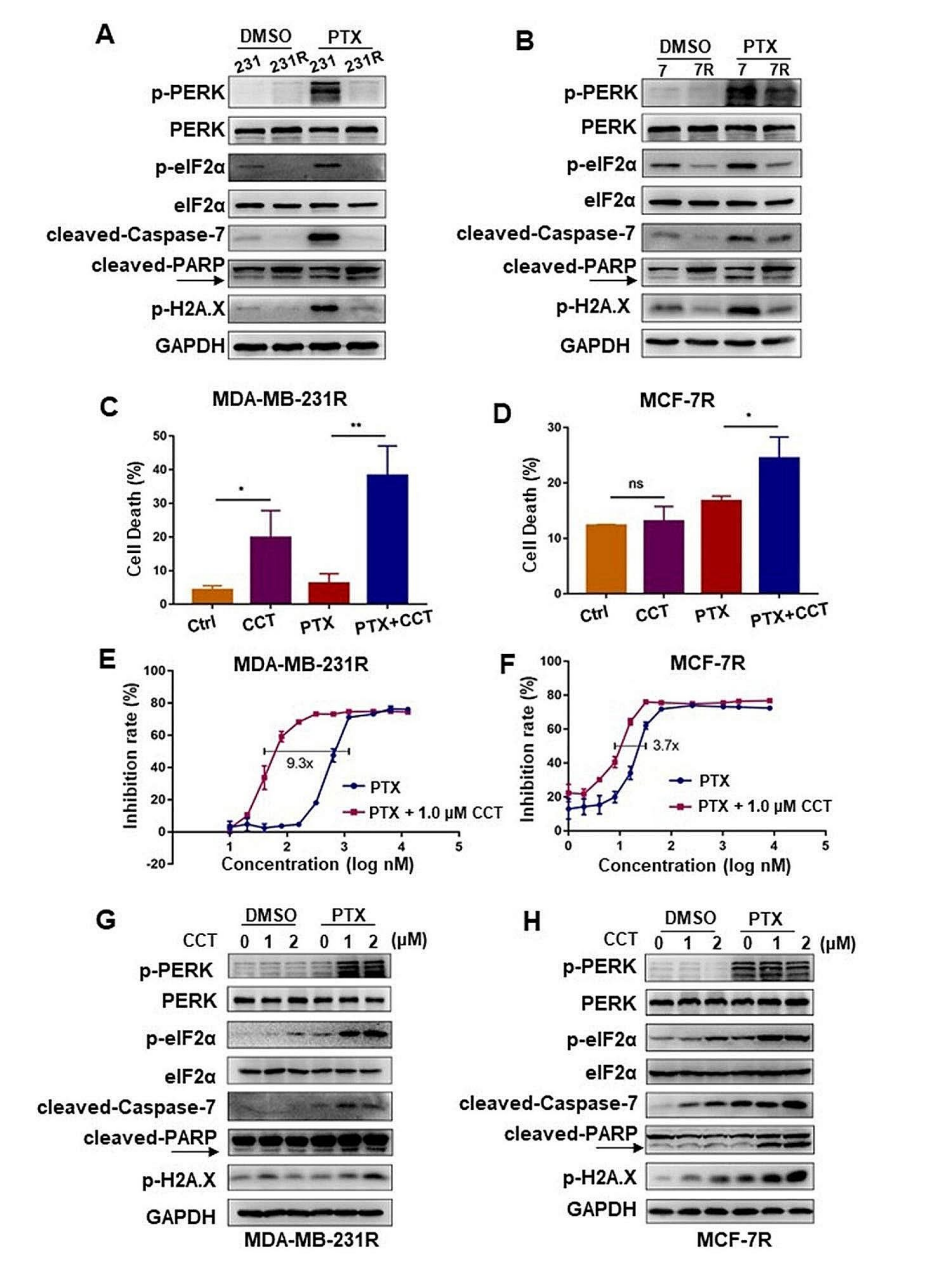
PTX combined with PERK agonist CCT effectively kills PTX-resistant breast cancer cells

Our results clearly demonstrated that the PERK/eIF2α axis could play a crucial role in taxane-induced tumor cell death. On the contrary, evidence suggests that PERK/eIF2α axis activation, especially PERK activation can positively enhance chemotherapy resistance [36–42]. To clarify this puzzle in breast cancer cells, we examined the expression of p-PERK and p-eIF2α in two PTX-resistant cells in comparison with their parental counterparts. Immunoblots showed that under PTX treatment, both p-PERK and p-eIF2α remained at low expression levels, followed by the low activation of apoptotic effectors in MCF-7R and MDA-MB-231R cells. Even under normal culture conditions, lower endogenous p-eIF2α expression level was found in MCF-7R and MDA-MB-231R cells than their parental counterparts (Fig. 5A, B). Thus, our results uncovered a decrease in eIF2α phosphorylation would be a novel hallmark of PTX resistance breast cancer cells.

Given the marked distinction of the p-PERK and p-eIF2α levels between PTX-resistant cells and their parental counterparts, we sought to reverse PTX resistance by activating PERK/eIF2α axis in the PTX-resistant cells with a selective PERK activator, CCT020312 (CCT), which contributes the eIF2α phosphorylation [43]. Flow cytometry of Annexin V-FITC/PI double staining indicated that in combination with CCT, PTX in turn strikingly promoted cell apoptosis in both MDA-MB-231R and MCF-7R cells, while CCT alone showed no notable cytotoxic side effect (Fig. 5C, D, Fig S5A, B). The IC_50_ of CCT is shown in the supplemental Table 1. Likewise, cell viability assay (CCK-8) showed that compared with PTX alone treatment, CCT combined with PTX exhibited significant inhibitory effects on the growth of MDA-MB-231R and MCF-7R cells (Fig. 5E, F) with an obvious reduction of IC_50_ of PTX (Table 1). Meanwhile, compared with the treatment of PTX or CCT alone, the combination of CCT with PTX markedly increased p-eIF2α levels in MDA-MB-231R and MCF-7R cells (Fig. 5G, H), indicating the positive correlation of p-eIF2α levels and cytotoxic effect of PTX.

**Table 1:** IC_50_ (nM) of PTX alone or in combination with CCT against breast cancer cells and their PTX-resistant (R) counterparts

Cell lines	PTX alone	PTX with CCT
MDA-MB-231R	692.76	74.26
MDA-MB-231	2.40	2.26
MCF-7R	49.24	13.40
MCF-7	3.59	5.53

However, in PTX-sensitive MDA-MB-231 and MCF-7cells, no notable change was observed in cell viability (Fig S6A, B) nor apoptosis percentage (Fig S6C-F) under PTX and CCT combination treatment. Immunoblots also showed no obvious increase in p-eIF2α level under combination treatment with CCT. In parallel, PTX alone induced enough eIF2α phosphorylation in sensitive cells (Fig S6G, H). Together, these results further confirmed the important and synergic role of phosphorylation of eIF2α in sensitizing tumor cells to PTX treatment.

(A-B) Immunoblots of PERK/eIF2α axis and apoptosis-related protein in MDA-MB-231, MDA-MB-231R cells (A) and MCF-7, MCF-7R cells (B) treated with 100 nM and 10 nM PTX for 24 h, respectively. (C-D) Quantitative and statistical analyses of cell death (Annexin V positive) of MDA-MB-231R (C) cells treated with 300 nM PTX or MCF-7R (D) with 30 nM PTX combined with 2µM CCT for 24 h, respectively. All assays were performed in triplicates. * *p* < 0.05;** *p* < 0.01; ns, no statistical significance. (E-F) CCK-8 assay measuring cell viabilities of MDA-MB-231R cells (E) or MCF-7R (F) treated with PTX at the indicated concentrations combined with 1 µM CCT for 96 h. All assays were performed in triplicates. (G-H) Immunoblots of PERK/eIF2α axis and apoptosis-related protein in MDA-MB-231R cells treated with 300 nM PTX (G) or MCF-7R cells with 30 nM PTX (H) combined with CCT at the indicated concentrations for 24 h.

### Combination with PERK activator efficiently inhibits PTX-resistant tumor growth

**Fig. 6:**
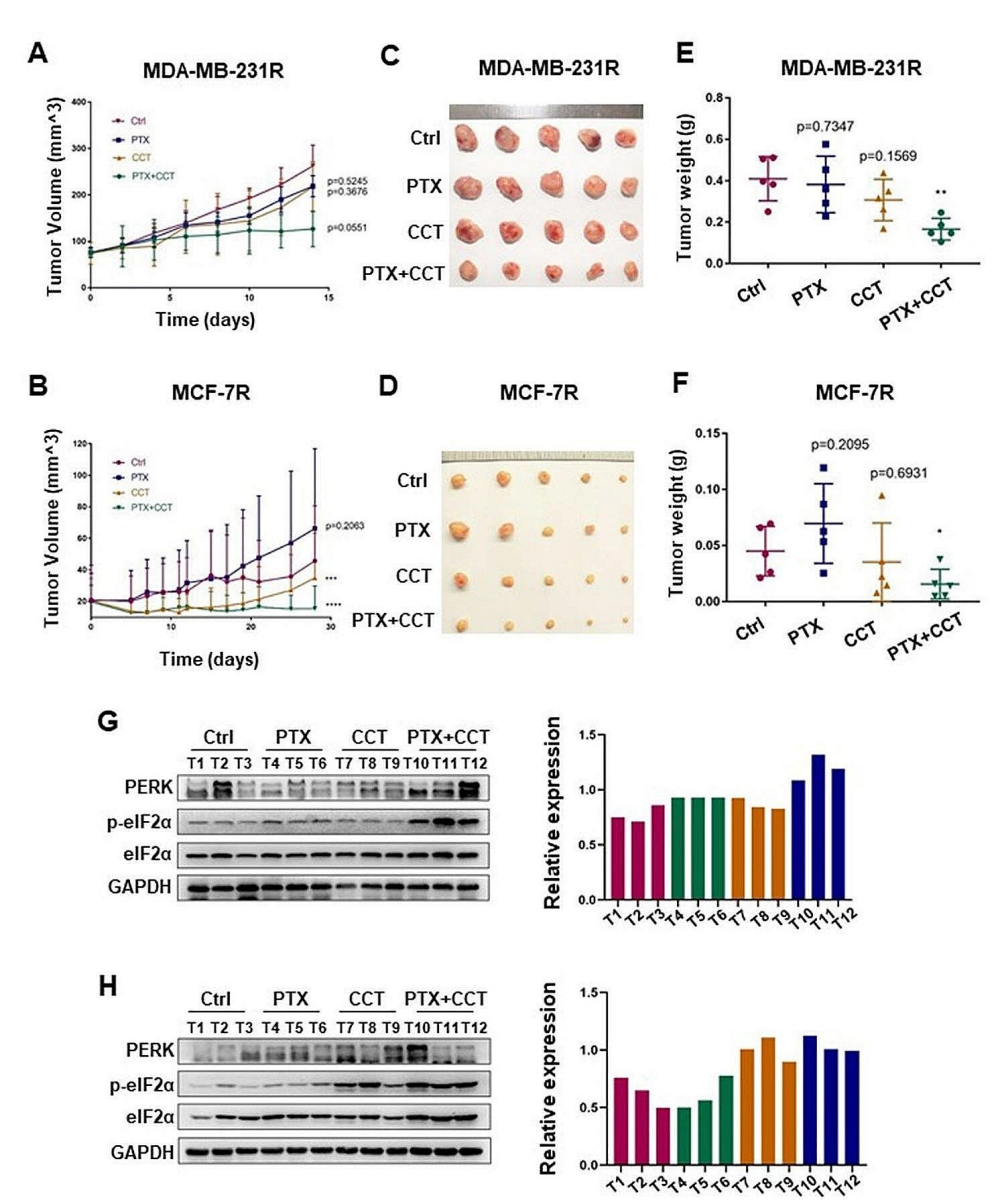
PTX combined with CCT synergically represses the subcutaneous PTX-resistant tumor growth

To further validate the practicability of combined administration of PTX with CCT for PTX-resistant tumor therapy, we established two subcutaneous xenograft tumor models by injecting MDA-MB-231R and MCF-7R cells into the breast pad of nude mice, respectively. Drugs were given according to schedules indicated in the supplemental Tables 2 and Table 3 Solvents of PTX and CCT were used as vehicle control. Compared with PTX alone treatment, the combined treatment of PTX with CCT efficiently inhibited the tumor growth of MCF-7R and MDA-MB-231R inoculated xenografts in tumor volume (Fig. 6A, B) and the net weight of the dissected tumors (Fig. 6C-F). For MCF-7R tumor xenografts, they showed smaller tumor volume at the end of treatment, which might result from the relatively earlier treatment time point and slower tumor growth rate.

To verify the relationship between the anti-neoplastic effect of CCT combined treatment and the phosphorylation level of eIF2α, we detected the expression of p-eIF2α by immunoblots in the harvested xenografted tumors. Results showed that the combination treatment groups of both MDA-MB-231R and MCF-7R tumors showed the strongest expression of p-eIF2α, indicating the increased phosphorylation of eIF2α would indeed potentiate the therapeutic effect of PTX on MDA-MB-231R and MCF-7R tumor xenografts (Fig. 6G, H). Thus, our results demonstrated that combined administration of PTX and CCT exhibits synergic and promising therapeutic potential in PTX-resistant breast tumors.

(A-B) Tumor growth of MDA-MB-231R and MCF-7R xenografts treated with PTX or CCT alone or in combination for 2 weeks (MDA-MB-231R) and 4 weeks (MCF-7R). Tumor volumes were measured 3 or 4 times a week. (C-F) The tumor was dissected at the endpoint of treatment and tumor weight was quantified. (G, H) Immunoblots of PERK/eIF2α axis and the relative expression of p-PERK/ p-eIF2α in the indicated mice tumor tissues. All statistical analyses are shown as *P < 0.05; **P < 0.01; ***P < 0.001; ****P < 0.0001.

## Discussions

The role of PERK/eIF2α axis activation in the regulation of cell death is elusive. On the one hand, the activation of PERK/eIF2α axis has been considered as a protective mechanism by upregulating serval stress-related proteins, such as NRF2, ATF4, GRP78, to reduce the ROS level, decrease the production of unfolded or misfolded proteins and finally restore the intracellular homeostasis [41, 42, 44–46]. However, it is also reported that PERK/eIF2α axis activation contributes to cell death event [47–50]. In this study, we demonstrated that the activation of the PERK/eIF2α axis is a pivotal prerequisite of taxanes to initiate cancer cell apoptosis, which is independent of the well-known microtubule polymerization-dependent manner.

ER stress response usually happens upon outside stresses and consequently activates the UPR (unfolded protein response) for protein homeostasis of cells. PERK, IRE1α, and ATF6 are the three main molecular sensors or indicators of UPR. In our study, we detected the activation status of these three indicators in MCF-7 cells upon PTX treatment in a time-course manner and found only PERK is obviously activated (Fig S1A). In addition, p-IRE1α was not triggered even by high dosage of taxanes at 10 µM in HCT-116 cells. While it showed a marginal change in MDA-MB-231 cells (Fig S1B-D). These results implied that ER stresses induced by taxanes is cell-type and dose-dependent. Nevertheless, PTX induced PERK/eIF2α most obviously in breast cancer cells and the mechanism by which needed to be explored in future work.

The mechanism of PERK/eIF2α axis activation on apoptosis induction is complicated. In this study, we observed that CHOP is upregulated under PTX treatment. Generally, CHOP is regarded as the downstream marker of the PERK/eIF2α axis which triggers the intrinsic apoptotic pathway through the regulation of Bcl-2 protein family [51, 52]. Bax and Bak may function as executioners in ER stress-mediated apoptosis [53, 54]. In our study, the expression of Bax is up-regulated along with CHOP, but the regulation details still need to be dissected.

DNA damage can be both the cause and the result of apoptotic cell death. Some DNA-damaging reagents, such as cisplatin, 5-fluorouracil, doxorubicin, can directly bind to and destory the intracellular DNA, leading to replication stress and subsequent cell death via apoptosis [55, 56]. Meanwhile, DNA fragmentation by the caspase-activated DNase (CAD) also contributes to massive DNA damage during apoptosis, accompanying particularly extensive p-H2A.X [57, 58]. It has been reported that mitosis arrest induced by taxanes leads to DNA damage [59]. Here we found the positive correlation between PERK activation and p-H2A.X level in PTX-treated cells. Therefore, the PERK/eIF2α axis activation could be involved in promoting DNA damage to simultaneously render the cytotoxicity of PTX along with microtubule polymerization at particular content.

β-tubulin is the direct target of taxanes and microtubule polymerization is the first cellular dysfunction discovered after the treatment of taxanes [1, 9]. Therefore, we tried to explore the relationship between PERK/eIF2α axis activation and microtubule polymerization. Our data showed that the ultra-low amount of PTX (picomolar, pM) can induce PERK/eIF2α activation without observable microtubule polymerization, indicating the activation of the PERK/eIF2α axis is independent on microtubule polymerization upon taxane stimulation. To thoroughly verify their relationship, the usage of cell with mutated β-tubulin that is unable to bind with taxanes is a straightforward way [60–62]. Meanwhile, the derivatives of PTX that contatin similar molecular structure to PTX but cannot bind to β-tubulin should be included [63].

Taxane resistance is a critical barrier to its effective and long-term usage, but the underlying causes are multiple and intriguing, including tubulin modifications, P-gp efflux overexpression, NF-κB activation [10, 64, 65], and so on. Although efforts expensed to explore the treatment targeting these factors, few strategies have been shown helpful in clinical treatment [66–68]. Thus, additional efforts are needed to better understand the molecular mechanisms underlying taxane resistance. Here we demonstrated the inactivation of PERK/eIF2α axis may be another important cause of PTX resistance, which should be verified with more types of PTX-resistant cells.

Overall, we clearly showed that activation of the PERK/eIF2α axis is another critical upstream event to trigger cell apoptosis, besides microtubule polymerization upon taxane stimulation, and reactivation of PERK/eIF2α activation would be a promising strategy to sensitize breast tumors to PTX treatment.

### Electronic supplementary material

Below is the link to the electronic supplementary material.

Supplementary Material 1

## Data Availability

No datasets were generated or analysed during the current study.
