# Supplementary material for: Activation of the PERK/eIF2α axis is a pivotal prerequisite of taxanes to cancer cell apoptosis and renders synergism to overcome paclitaxel resistance in breast cancer cells
Source: Cancer Cell Int. 2024 Jul 17;24:249. doi: 10.1186/s12935-024-03443-w (PMC11256575; doi:10.1186/s12935-024-03443-w)
Supplement: Supplementary file 1 — Supplementary Material 1 [file 12935_2024_3443_MOESM1_ESM.pdf]

Supplemental Figures and Tables

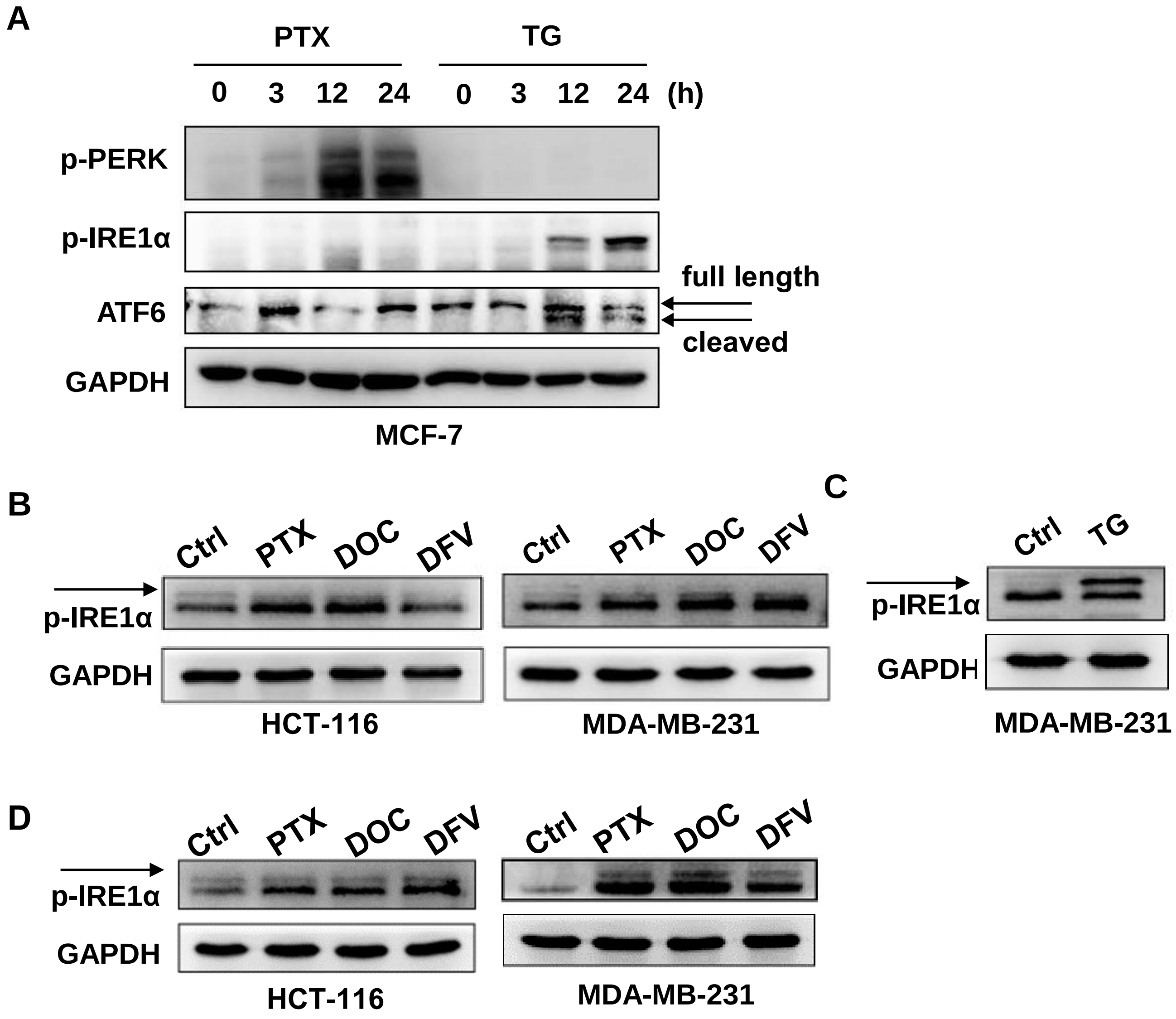

Figure S1. Incomplete activation of UPR signaling under Taxanes treatment.

(A) Immunoblots of the dynamic expression of the activated molecular sensors of UPR in MCF-7 cells treated with 100 nM PTX at indicated time points. 5 μM Thapsigargin (TG) as positive control. (B) Immunoblots of p-IRE1α in HCT-116 cells and MDA-MB-231 cells treated with 100 nM taxanes (PTX, DOC, DFV-OTX) for 24 h. (C) MDA-MB-231 cells treated with 10 μM Thapsigargin (TG) as positive control. (D) Immunoblots of p-IRE1α in HCT-116 cells and MDA-MB-231 cells treated with 10 μM taxanes (PTX, DOC, DFV-OTX) for 24 h.

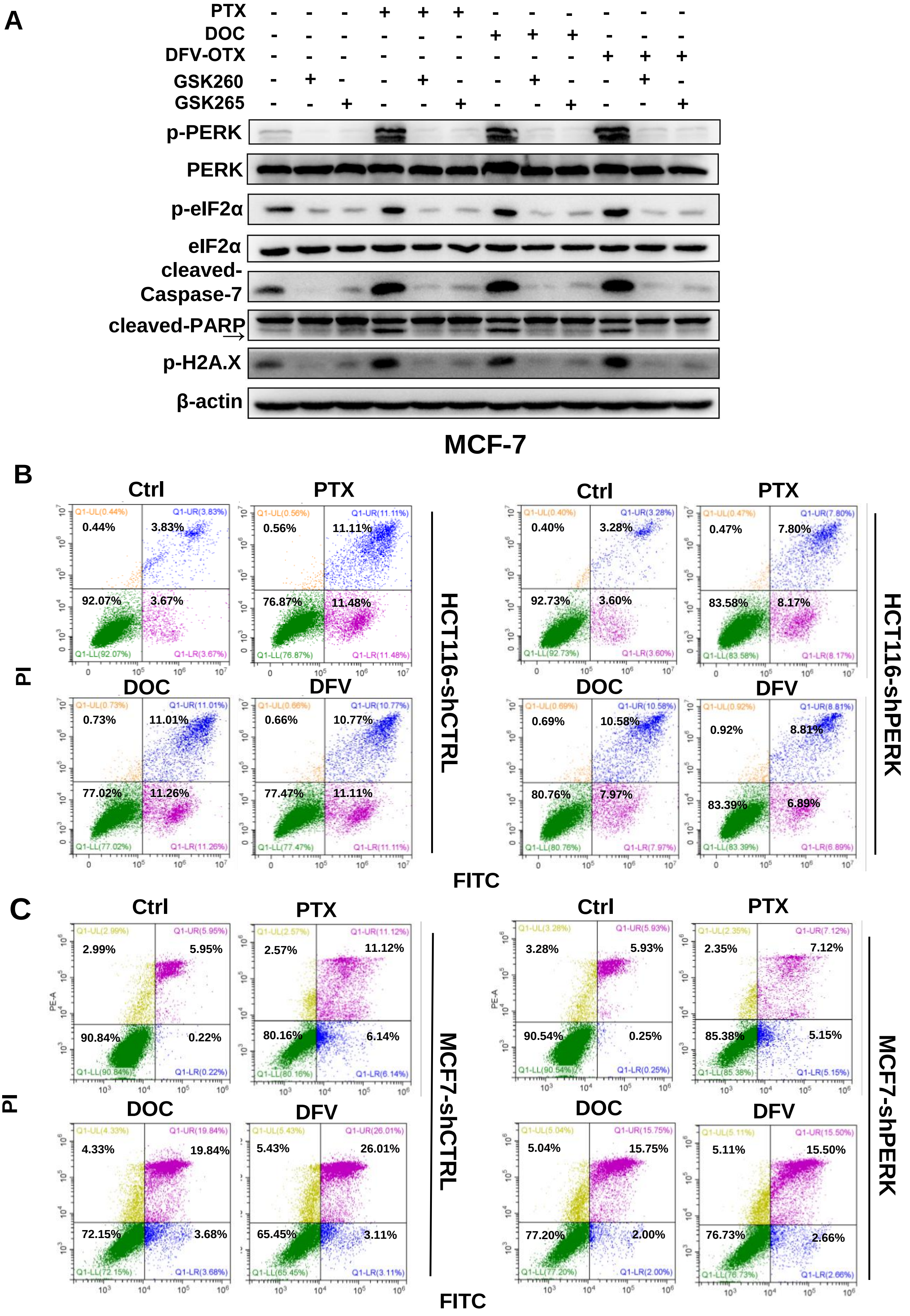
A
PTX
DOC
DFV-OTX
GSK260
GSK265
p-PERK
PERK
p-eIF2α
eIF2α
cleaved-Caspase-7
cleaved-PARP
p-H2A.X
β-actin
MCF-7
B
Ctrl
PTX
DOC
DFV
PI
FITC
HCT116-shCTRL
HCT116-shPERK
0.44%
3.83%
92.07%
3.67%
0.56%
11.11%
76.87%
11.48%
0.73%
11.01%
77.02%
11.26%
0.66%
10.77%
77.47%
11.11%
0.40%
3.28%
92.73%
3.60%
0.47%
7.80%
83.58%
8.17%
0.69%
10.58%
80.76%
7.97%
0.92%
8.81%
83.39%
6.89%
C
Ctrl
PTX
DOC
DFV
PI
FITC
MCF7-shCTRL
MCF7-shPERK
2.99%
5.95%
90.84%
0.22%
2.57%
11.12%
80.16%
6.14%
4.33%
19.84%
72.15%
3.68%
5.43%
26.01%
65.45%
3.11%
3.28%
5.93%
90.54%
0.25%
2.35%
7.12%
85.38%
5.15%
5.04%
15.75%
77.20%
2.00%
5.11%
15.50%
76.73%
2.66%

Figure S2. Inhibition of the PERK/eIF2α axis blocks taxane-induced cell death.

(A) Immunoblots of PERK/eIF2α axis and apoptosis-related protein in MCF-7 cells treated with 100 nM taxanes combined with 10 μM GSK265 or GSK260 for 24 h. (B) Annexin V-FITC/PI double-staining of HCT-116 cells with PERK knockdown treated with 100 nM taxanes for 24 h. (C) Annexin V-FITC/PI double-staining of MCF-7 cells with PERK knockdown treated with 100 nM taxanes for 24 h.

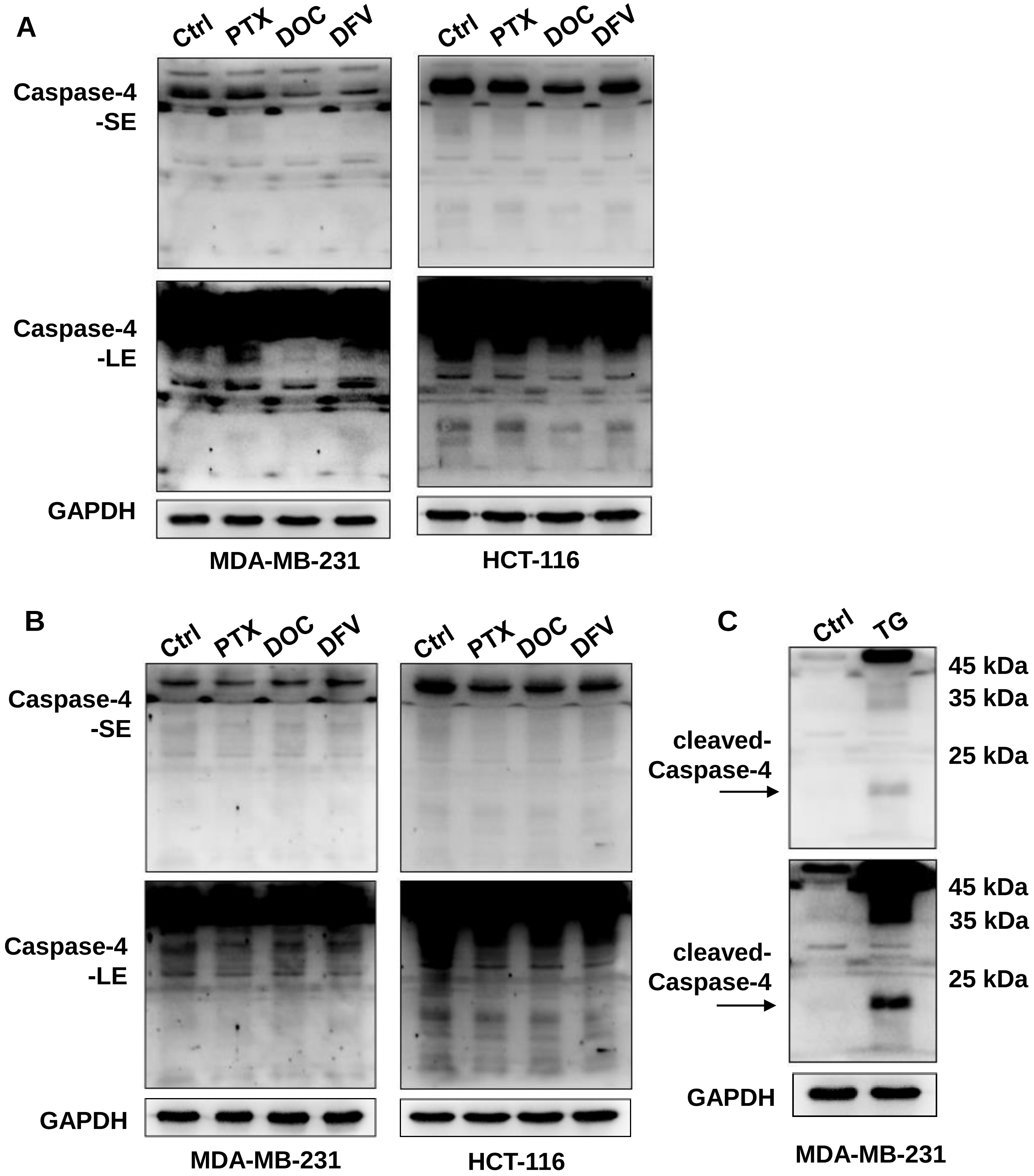

Figure S3. PERK/eIF2α axis triggers downstream cell apoptotic events bypass Caspase-4. Immunoblots of Caspase-4 in HCT-116 and MDA-MB-231 cells treated with 100 nM taxanes (A) or 10 μM taxanes (B) for 24 h. SE and LE represent short exposure and long exposure, respectively. (C) MDA-MB-231 cells were treated with 10 μM Thapsigargin (TG) as positive control.

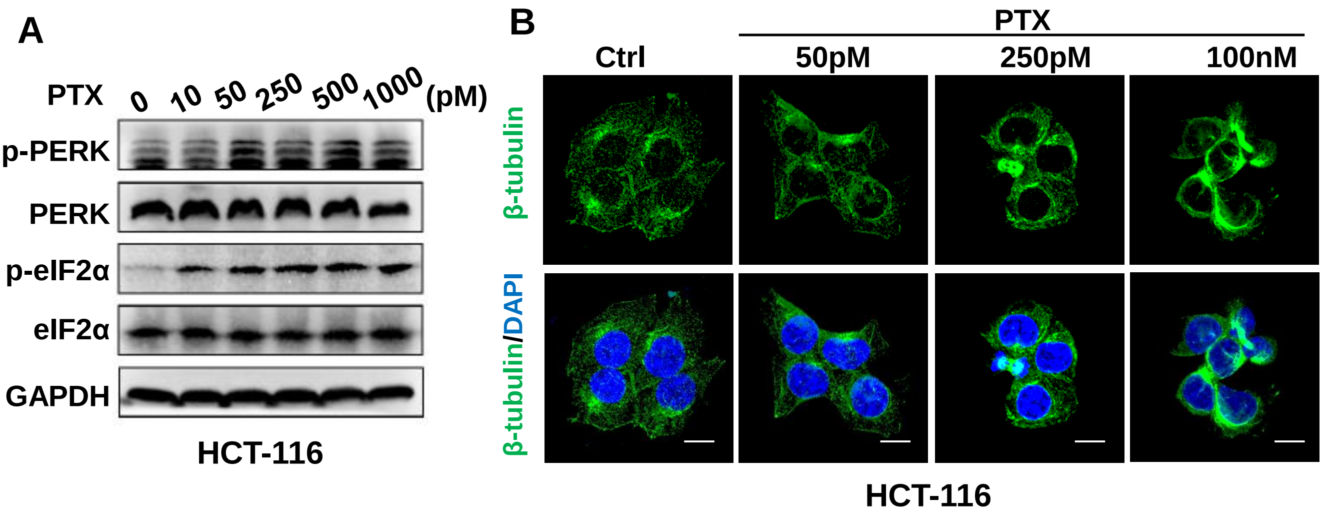

Figure S4. Activation of PERK/eIF2α axis along with no microtubule polymerization under ultra-low concentration of PTX treatment in HCT-116 cells.

(A) Immunoblots of PERK/eIF2α axis in HCT-116 cells treated with PTX at ultra-low concentration as indicated. (B) Confocal microscopy of microtubule distribution in HCT-116 cells treated with 50 pM or 250 pM PTX for 24 h. Treatment of 100 nM PTX is the positive control of microtubule polymerization.

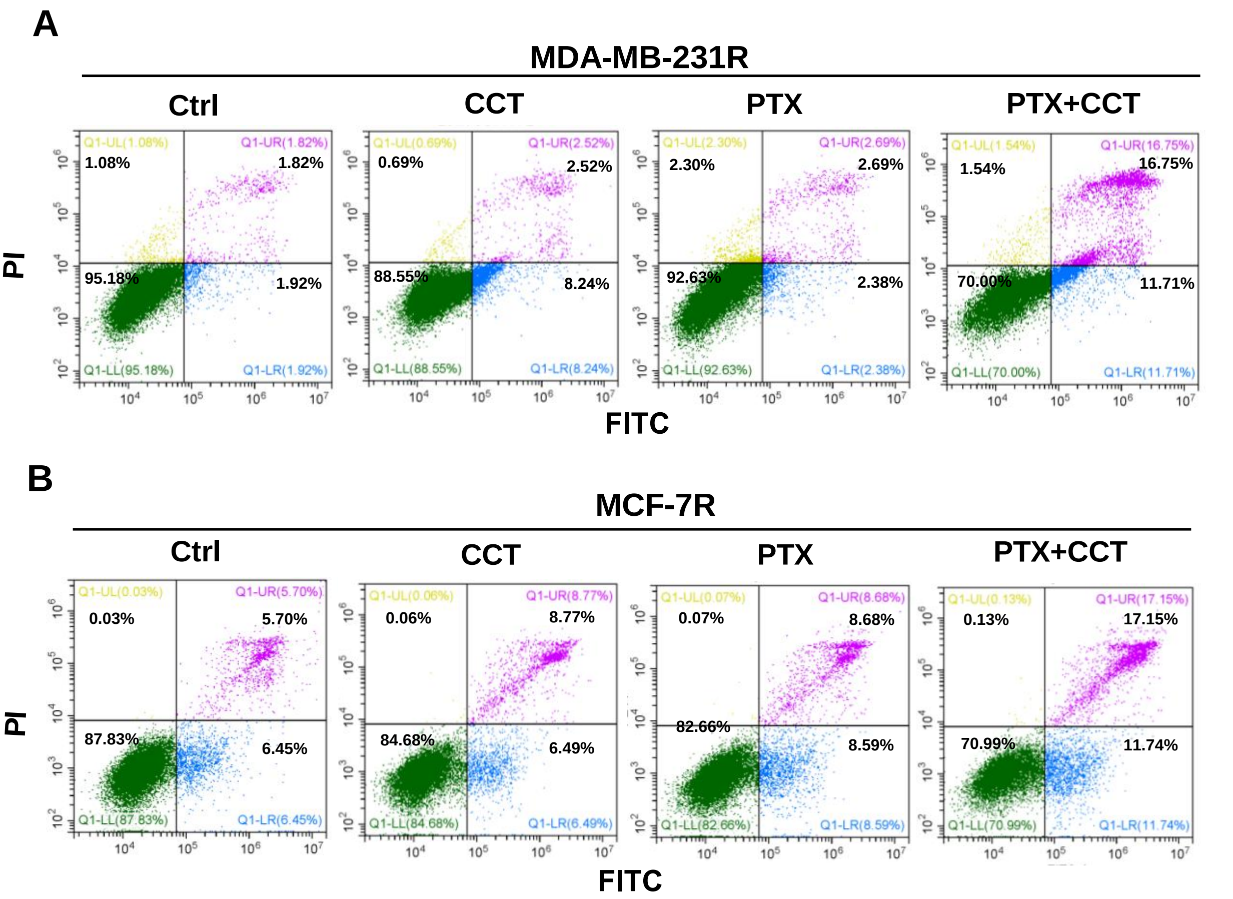

Figure S5. Cell death of paclitaxel-resistant breast cancer cells assessed by flow cytometry.

(A) Annexin V-FITC/PI double-staining of MDA-MB-231R cells treated with 300 nM PTX combined with 2 μM CCT for 24 h.

(B) Annexin V-FITC/PI double-staining of MCF-7R cells treated with 30 nM PTX combined with 2 μM CCT for 24 h.

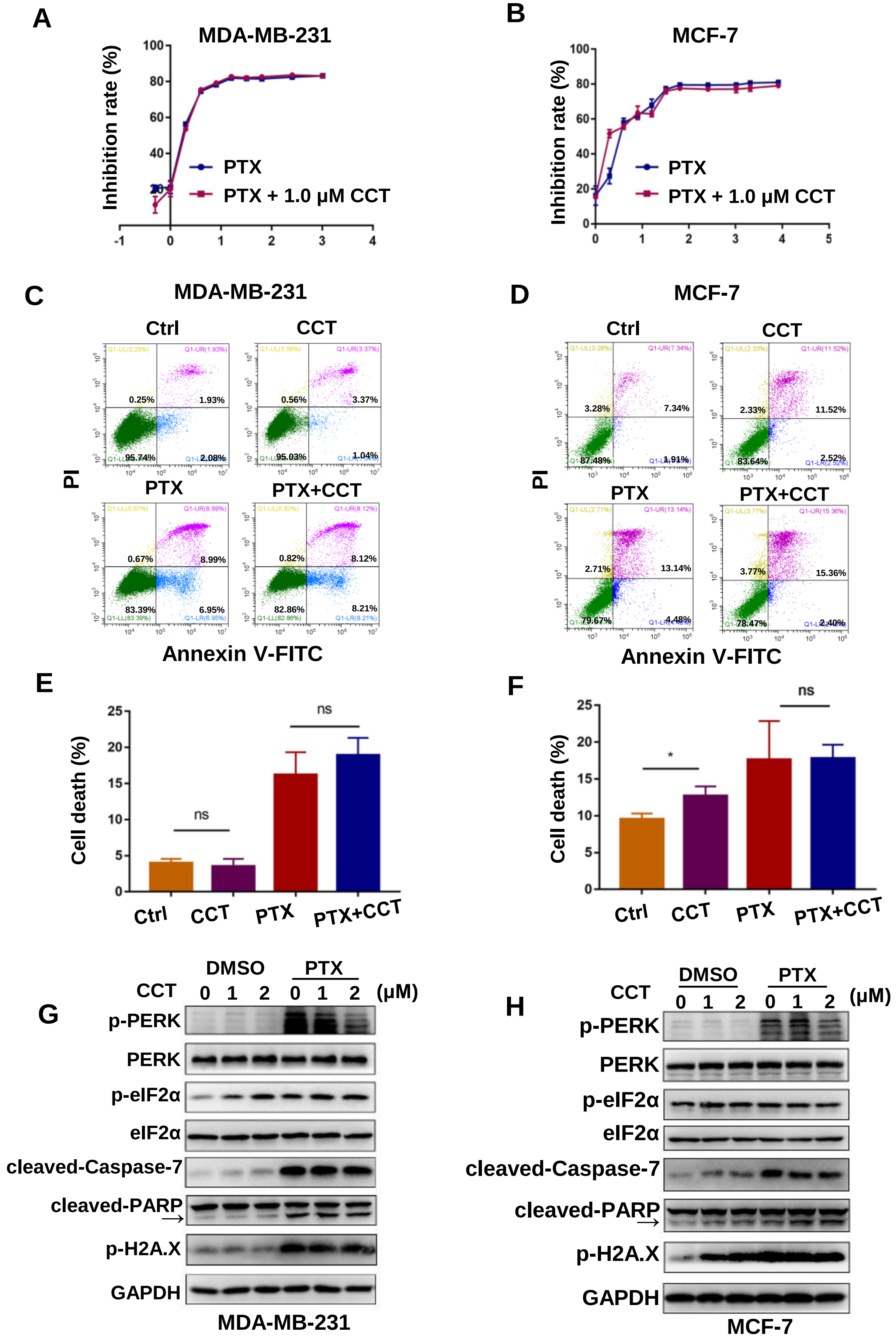
A
MDA-MB-231
Inhibition rate (%)
PTX
PTX + 1.0 μM CCT
B
MCF-7
C
MDA-MB-231
Ctrl
CCT
PTX
PTX+CCT
PI
Annexin V-FITC
D
MCF-7
E
Cell death (%)
ns
Ctrl
CCT
PTX
PTX+CCT
F
*
G
DMSO
PTX
CCT
0 1 2 0 1 2 (μM)
p-PERK
PERK
p-eIF2α
eIF2α
cleaved-Caspase-7
cleaved-PARP
p-H2A.X
GAPDH
MDA-MB-231
H
MCF-7

Figure S6. PTX combined with CCT showed no synergistic effect on parental MCF-7 and MDA-MB-231 cells.

(A-B) CCK-8 assay measuring cell viability of MDA-MB-231 cells (A) and MCF-7 cells (B) treated with PTX at the indicated concentrations combined with 1 μM CCT for 96 h. (C-D) Annexin V FITC/PI double staining of MDA-MB-231 cells (C) treated with 300 nM PTX or MCF-7 (D) with 30 nM PTX combined with 2μM CCT for 24 h, respectively. (E-F) Statistical analysis of the Annexin V positive percentage of MDA-MB-231 and MCF-7 cells in (C-D). All assays were performed in triplicates. ns: no statistical significance; * $p<0.05$. (G-H) Immunoblots of PERK/eIF2α axis and apoptosis-related protein in MDA-MB-231 cells (G) treated with 300 nM PTX or MCF-7 (H) with 30 nM PTX combined with CCT at the indicated concentrations for 24 h, respectively.

Table S1. Cytotoxicity (IC_{50} μM) of CCT in PTX -resistant and -sensitive breast cancer cells

Cell lines	IC_{50} of CCT (μM)
MDA-MB-231R	2.78
MDA-MB-231	3.38
MCF-7R	1.65
MCF-7	3.20

Table S2. Treatment regimen for assessing the therapeutic efficacy of PTX combined with CCT on MDA-MB-231R derived xenograft tumors.

	PTX	CCT	Combo	
Time (day)	PTX (mg/kg)	CCT (mg/kg)	PTX (mg/kg)	CCT (mg/kg)
D1		15		15
D2	30	15	30	15
D5		15		15
D6	30	15	30	15
D10		15		15
D11	30	15	30	15
D14				
Total	90	90	90	90

Table S3. Treatment regimen for assessing the therapeutic efficacy of PTX combined with CCT on MCF-7R derived xenograft tumors.

	PTX		Combo	
Time (day)	PTX (mg/kg)	CCT (mg/kg)	PTX (mg/kg)	CCT (mg/kg)
D1		15		15
D2	15	15	15	15
D5		15		15
D6	15	15	15	15
D12		10		10
D13	10	10	10	10
D20		10		10
D21	10	10	10	10
D30				
Total	50	100	50	100
